# Dataset on the effect of foliar application of different concentrations of silicon dioxide and organosilicon compounds on the growth and biochemical contents of oak leaf lettuce (Lactuca sativa var. crispa) grown in phytotron conditions

**DOI:** 10.1016/j.dib.2021.107328

**Published:** 2021-08-26

**Authors:** Ali J. Othman, Ludmila G. Eliseeva, Nazirya A. Ibragimova, Valeriy N. Zelenkov, Vyacheslav V. Latushkin, Denitsa V. Nicheva

**Affiliations:** a Department of Commodity Research and Commodity Expertise, Plekhanov Russian University of Economics, Moscow, Russian Federation; b All-Russian Scientific Research Institute Vegetable Growing-The Branch of FSBSI “Federal Scientific Centre of Vegetable Growing, Moscow, Russian Federation; c Independent NPO Institute for Socio-Economic Strategies and Development Technologies (Institute for Development Strategies), Moscow, Russian Federation; d Acad. E. Budevski, Institute of Electrochemistry and Energy Systems, Bulgarian Academy of Sciences, Bulgaria

**Keywords:** Lactuca sativa var. crispa, Antioxidants capacity, Phenolic content, Organosilicon, Silicon dioxide, Phytotron, Capillary zone electrophoresis

## Abstract

This article presents comparative data regarding the effect of foliar application of silicon dioxide and organosilicon compounds on soilless-grown Oak leaf lettuce (Lactuca sativa var. crispa). Data were derived from dry and fresh samples. Total nitrogen, total antioxidants capacity, total phenolic content, ascorbic acid, total pigments concentrations and growth parameters varied in response to the concentrations of the used preparations (silicon dioxide and organosilicon compounds). Capillary zone electrophoresis, spectrophotometry and coulometric analyzer were the principal involved methods. Data of total phenolic content, antioxidants capacity and ascorbic acid concentrations can provide physiological health benefits as functional foods along with an insight to plant stress physiology. Chlorophyll a and b concentrations, nitrogen content, dry matter content, plant height and fresh weights contribute to the understanding of physiological and biometric plants growth parameters.

## Specifications Table

**Table:** 

Subject	Agricultural and Biological Sciences
Specific subject area	Plant Nutrition, Horticultural Science, Hydroponics, Silatranes, Silicon dioxide, Food analysis
Type of data	Table and raw data
How data were acquired	Capillary electrophoresis Capel m-105 (Lumex, Russian Federation) Coulometric analyzer EXPERT-006 (Econix-Expert, Russian Federation) Spectrophotometer (Shimadzu UV 2401pc, Japan) Express analysis tester (ECOVISOR, Russian Federation) Multi-detection microplate reader Synergy HT (Bio-Tek, USA) Moisture analyzer MX-50 (A & D, Japan)
Data format	Raw and analyzed data
Parameters for data collection	0.20 mm filter papers Centrifuge at speed of (10000, 13000, and 15000 rpm) Ultrasonic water bath 60 °C for 10 min Moisture analysing at 105 °C till constant weight Spectrophotometer at 470, 647 and 664 nm wavelengths Multi-detection microplate reader at 725 nm wavelength Capillary electrophoresis at 254 nm wavelengths Infused silica capillary length: 50 cm, diameter: 50 µm
Description of data collection	The data on total chlorophyll and carotenoids concentrations were obtained by spectrophotometry according to Lichtenthaler and Wellburn [1,2]. The data on total phenolic content were analyzed by the Folin–Ciocalteu colorimetric method using gallic acid as a standard as described by [3]. The data on total antioxidant capacity were obtained by coulometric analysis method using electrogenerated bromine radicals as described by Lapin A, Gorbunova, and Zelenkov [4]. The data on ascorbic acid content were obtained by capillary zone electrophoresis method [5]. The data on leaf total nitrogen were acquired according to the Kjeldahl method [6]
Data source location	Russian economic University. G. V. Plekhanova, Stremyanny avenue, 36, Moscow, 115093, Russia Federation Institution: Plekhanov Russian University of economics City/Town/Region: Moscow Country: Russian Federation Latitude and longitude for collected samples: 55.7273504, 37.625481
Data accessibility	With the article Raw data (Supplementary material) Mean and standard error of the experimental data are available in this article in Table 1.
Related research article	

## Value of the Data

• To our knowledge this dataset is the first trial regarding the effect of foliar treatments of 1-ethoxysilatrane (an organosilicon based compound) on the bioactive components that form most of the nutritional value of lettuce (Lactuca sativa L.).
• These data are useful for leafy vegetables growers and plant factories by providing additional solution to increase the yield of the crops.
• These data might be useful in the nutritional optimization of dietary intake, especially when antioxidants and phenols constitute a serious consideration (e.g. antioxidants and cardiovascular diseases [7]).
• Data on nitrogen accumulation and total chlorophyll content provides an additional information along with plants growth parameters in assessing the activation of physiological activities in the plants.
• These data show the effective concentration of silicon dioxide and 1-ethoxysilatrane on oak leaf lettuce nutritional content after the application of foliar treatment.
• Lowest concentration of 1-ethoxysilatrane (ES4) resulted with the highest accumulation of TAC, Total carotenoids, total phenols, fresh weight and lettuce plants heights of lettuce plants and number of leaves developed.

## 1 Data Description

The foliar application of Si containing preparations can modulate the metabolism, bioactive components, nutritional profile, and quality of leafy vegetables [8,9]. 1-ethoxysilatrane is a newly emerging biologically active organosilicon compound belongs to Silatrans family [10]. The dataset contains analyzed data obtained by spectrophotometric, Kjeldahl, capillary zone electrophoresis, and coulometric analysis methods from fresh and dried samples of soilless-grown oak leaf lettuce in closed climatic chamber (phytotron). Information provided about ascorbic acid, total nitrogen content, total antioxidant capacity, total phenolic content, total pigments content, and growth parameters of oak leaf lettuce cultivars as being affected by foliar treatments of organosilicon preparation and/or silicon dioxide are displayed in Table 1.

**Table 1:** Dry weight, total nitrogen, TAC, total chlorophyll, total carotenoids, ascorbic acid, total phenols, fresh weight, plant's height, and number of leaves of lettuce plants grown hydroponically in phytotron (ISR 0.1) after foliar treatments with different concentrations of 1-ethoxysilatrane and silicon dioxide.

Treatment	Dry weight (%)	Total N (mg kg-1 FW)	TAC (mg R.E/g)	Total chlorophyll (mg/g) FW	Total Carotenoids (mg/g)	Ascorbic acid (mg/100g)	Total phenols (mg GAE/g FW)	FW (grams)	Height (cm)	Number of leaves
Control	7.38 ± 0.17 g	49.68 ± 1.59 e	10.01 ± 0.34 c	17.63 ± 0.36 g	6.47 ± 0.13c	5.49 ± 0.10 e	28.73 ± 1.83 d	36.18 ± 2.86 c	16.31 ± 0.71 cd	17.16 ± 1.57 c
AS1	8.05 ± 0.05 d	45.60 ± 1.74 f	17.78 ± 0.89 a	25.98 ± 0.22 bc	8.34 ± 0.49 a	7.75 ± 0.11 b	54.54 ± 1.90 b	46.33 ± 1.03	24.92 ± 1.47 a	19.33 ± 0.75 b
AS2	7.6 ± 0.1 f	58.71 ± 1.09 d	14.56 ± 0.14 b	25.41 ± 0.07 c	7.04 ± 0.04 bc	6.09 ± 0.07 c	44.66 ± 2.40 c	45.27 ± 1.82 b	17.55 ± 1.39 c	17.33 ± 0.75 c
AS3	8.85 ± 0.08 b	61.66 ± 0.82 c	9.72 ± 0.43 c	26.50 ± 0.12 ab	6.37 ± 0.17 c	5.09 ± 0.05 f	26.86 ± 0.66 d	28.40 ± 1.71 d	14.68 ±0.60 d	13.01 ± 0.57 d
AS4	9.17 ± 0.15 a	76.30 ± 1.45 a	9.69 ± 1.26 c	27.08 ± 0.26 a	6.98 ± 0.26 bc	5.09 ± 0.28 f	24.27 ± 1.52 e	29.08 ± 0.87 d	12.73 ± 1.14 e	12.83 ± 0.69 d
ES1	7.40 ± 0.11 g	48.31 ± 2.01e	9.43 ± 0.14 c	17.97 ± 0.17 g	6.59 ± 0.11 c	5.72 ± 0.13 d	28.94 ± 1.58 d	36.24 ± 2.73 c	16.93 ± 1.04 c	17.16 ± 1.46 c
ES2	7.79 ± 0.05 e	46.28 ± 1.21 f	9.25 ± 0.08 c	19.96 ± 0.29 f	6.96 ± 1.26 bc	5.13 ± 0.12 f	27.47 ± 0.96 d	36.15 ± 2.64 c	17.25 ± 0.73 c	16.66 ± 0.75 c
ES3	7.99 ± 0.18 d	71.86 ± 0.92 b	15.62 ± 0.19 b	21.38 ± 1.05 e	8.07 ± 0.53 ab	7.77 ± 0.10 b	44.52 ± 2.72 c	43.83 ± 1.13 b	22.1 ± 1.60 b	22.33 ± 1.37 a
ES4	8.27 ± 0.12 c	77.95 ± 1.87 a	17.98 ± 0.72 a	22.63 ± 0.66 d	9.13 ± 0.14 a	7.94 ± 0.25a	59.12 ± 1.77 a	54.09 ± 3.36 a	23.86 ± 2.18 a	22.66 ± 1.59 a
N*	6	6	6	3	3	6	6	6	6	6

AS1, AS2, AS3, AS4, Silicon dioxide 50 mg.L^−1^, 100 mg.L^−1^, 200 mg.L^−1^, 333 mg.L^−1^, respectively.

ES1, ES2, ES3, ES4, 1-ethoxysilatrane 5.10^−4^ ml. L^−1^, 10^−3^ ml. L^−1^, 5.10^−3^ ml. L^−1^, 10^−2^ ml. L^−1^, respectively.

N*: number of repetitions. Different letters within each column indicate significant differences according to Duncan's test (P ≥ 0.05).

## 2 Experimental Design, Materials and Methods

### 2.1 Plant cultivation and experimental treatments

Oak leaf lettuce (Lactuca sativa var. crispa) Dubachek® (Agrofirma MoravoSeed, Mikulov, Czech Republic), was cultivated in a closed climatic growth chamber (phytotron ISR 0.1) conditions.

The experiment was carried out in 12th of March 2020 at the Plekhanov Russian university of economics in the department of goods commodity and expertise of goods products, Moscow-Russian Federation.

Plants were cultivated hydroponically on media substrate based on mineral wool placed in small plastic pots, three seeds were sown in each growing pot and cultivated for 34 days.

Oak leaf lettuce plants were drip-irrigated with a 0.25% Pioneer^TM^ nutrient solution (Broested, Denmark). The following amounts of nutrients were available for watering in mg.L^−1^: N 145, P_2_O_5_ 41, K_2_O 275, Ca 100, Mg 24, S 30, Fe 0.94, Mn 0.14, B 0.16, Cu 0.03, Zn 0.13, Mo 0.03. The pH of the nutrient solution ranged between 5.5-5.8, and the electrical conductivity was maintained between 1.8-2.0 mS.cm^−1^. Foliar treatment with silica-based preparations preparation were applied one upon 14^th^ day after germination using gradual concentrations of amorphous silicon dioxide (AS) and 1-ethoxysilatrane (ES) solutions, where: AS1, AS2, AS3, AS4, 50 ml.L^−1^, 100 mg.L^−1^, 200 mg.L^−1^, 333 mg.L^−1^, respectively. And ES1, ES2, ES3, ES4 refers to 5.10-4 ml.L^−1^, 10-3 ml.L^−1^, 5.10-3 ml. L^−1^, 10-2 ml. L^−1^ respectively. To reduce the salt content from the tap water, we used a combination of distilled and tap water in a proportion of 70:30 respectively. Photoperiod of 16 h and 22/15°C (day/night) temperature was sustained along with PPFD of 244 µmol m^−2^ s^−1^. The experiment comprised of randomized complete block design (RCBD), replicated six times for each treatment.

### 2.2 Dry weight

Dry weight content was determined by drying the shoots of lettuce plants until constant weight was reached at temperature of 105 °C using a humidity analyzer (MX-50, A&D Co, Japan).

### 2.3 Total nitrogen content

Leaf plant tissues were dried at 70 °C for 72 h (until constant weight was reached), and then ground in a Wiley Mill before being used for the analysis.

Total nitrogen in the dried plant samples were determined by the acid digestion Kjeldahl method [6] involving two steps: (1) digestion of the sample to convert the *N* to ammonium; (2) determination of the ammonium in the digest. A 0.1 g of macerated dry plant sample was weighed into a 250 mL Kjeldahl flask and a tablet 1 g of digestion accelerator (selenium catalyst), was added to ensure promoting the conversion of N to ammonium (NH_4_^+^) followed by 7 mL of 96% concentrated H_2_SO_4_ and 5 ml of 32% H_2_O_2_. The mixture was gently stirred until the digest became clear. Kjeldahl flask was then cooled and its content transferred into a 100 mL volumetric flask with distilled water and quantitatively made up to volume. A 5 mL aliquot of the digest was taken into a Markham distillation apparatus.

Then 5 mL of 40% NaOH solution was added to the aliquot and the mixture distilled. The distillate was collected in 5 mL of 2% boric acid containing solution. Three drops of a mixed indicator containing methyl red and methylene blue were added to the distillate in a 50 mL Erlenmeyer flask and then titrated against 0.01 M HCl acid solution. The percent nitrogen was calculated from the titer value and then was converted to g of nitrogen per kg of dry weight.

### 2.4 Total phenols content

The total phenolic contents were analyzed using the modified Folin–Ciocalteu colorimetric method [3]. A 0.05 g of the dry sample was grounded for 5 min with 2 mL of ice-cold 95% methanol using ice-cold mortar and pestle. The mixture was centrifuged at a speed of 13,000 rpm for 8 min at room temperature. Afterwards, a 1 mL of supernatant was mixed with 2.5 mL of 10% (w/v) Folin–Ciocalteu reagent. After 5 min, 2.0 mL of (20%) Na_2_CO_3_ was subsequently added to the mixture and incubated at 45°C for 20 min with intermittent agitation. After 90 min of incubation in the dark at ambient temperature, the absorbance was read at A_765_ nm. Total phenolic content was expressed as mg gallic acid equivalents (GAE) per gram of dry mass of lettuce using gallic acid calibration curve (R^2^=0.968).

### 2.5 Ascorbic acid determination

The content of the free form of vitamin C (Ascorbic acid) was determined by capillary zone electrophoresis method [5] utilizing (Kapel 105M, Lumex, Russian Federation) under positive high voltage polarity, the internal diameter of the capillary 50 µm, total length 50 cm. We used 10 mM sodium tetraborate as a buffer pH 9.2. Samples injected applying a pressure of 450 mbar.s^−1^, Voltage: +20 kV, ascorbic acid was detected at A_254_ nm, at 24°C. A 5 g of fresh sample was diluted to 100 cm^3^ and well mixed for 10 min in the dark then it was filtered and placed in Eppendorf tubes and centrifuged twice under 15000 rpm for 8 min to avoid any impurities. The supernatant was replaced into the device for analysis.

### 2.6 Total antioxidant capacity

The total antioxidants capacity (AOC) in oak leaf lettuce samples were determined using galvanostatic coulometric analyzer (Econix-Expert, EXPERT-006, Russia) calibrated with ascorbic acid. The principle of analysis [4], [10] based on Faraday's law, according to which the mass of the analyte is determined by the amount of electricity spent on the reaction.

Five grams of lettuce sample was macerated and exactly 1 gram was transferred into the electrochemical cell of the device containing 50 ml of 0.2 M KBr in a 0.1 M H_2_SO_4_ and stirred automatically for 20 s before the electrolysis process began. A pair of generator electrodes and an indicator electrode used for bipotentiometric determination of the titration end point. Bromine was generated at a constant current of 50 mA during mixing time. The potential of the initial and end value of the indicator system were set on 200 mV, the value from which the electrolysis process initiates was set on 40 mV. The end point of titration was fixed when the initial value of the indicator potential was reached. During the reaction time, all substances with antioxidants properties reacted with the excess of the generated bromine. The device automatically filtered the bromine outflow, which was numerically equal to the number of antioxidants introduced in the aliquot. Results are expressed in mg.g^−1^ of fresh lettuce.

### 2.7 Total chlorophyll and carotenoids

Accurately weighted 0.5 g of fresh plant leaf sample and homogenized with 10 ml of 96% alcohol. The homogenized sample mixture was centrifuge for 10,000 rpm for 15 min at 40°C. The supernatant was separated and 0.5 mL of it was mixed with 4.5 mL of ethanol 96%. The solution mixture was analyzed for Chlorophyll-*a*, Chlorophyll-*b* and total carotenoids content in spectrophotometer (Shimadzu UV 2401pc UV-VIS, Japan). The method and equations used for calculating the concentration of pigments are those of Lichtenthaler and Wellburn [1,2].

### 2.8 Experimental data analysis

Data analysis was subjected to one-way analysis of variance (ANOVA) using the IBM SPSS 25.0 version software (SPSS Inc., Chicago, Ill., USA) and treatment means separation was done by Duncan's test at 5% level of significance in order to separate treatment means within each measured parameter.

## Ethics Statement

None.

## Declaration of Competing Interest

The authors declare that they have no known competing financial interests or personal relationships which have, or could be perceived to have, influenced the work reported in this article.

## Appendix Supplementary materials

Image, application 1
